# A retrospective analysis of acute kidney injury in children with post-COVID-19 multisystem inflammatory syndrome: insights into promising outcomes

**DOI:** 10.1186/s13052-024-01598-w

**Published:** 2024-02-05

**Authors:** Hanan El-Halaby, Riham Eid, Ahmed Elagamy, Ahmed El-Hussiny, Fatma Moustafa, Ayman Hammad, Mayada Zeid

**Affiliations:** 1 Pediatric Critical Care Unit, Mansoura University Children’s Hospital, Faculty of Medicine, Mansoura University, Mansoura, Egypt; 2 Pediatric Nephrology Unit, Mansoura University Children’s Hospital, Faculty of Medicine, Mansoura University, Mansoura, Egypt; 3 Department of Pathology, Faculty of Medicine, Mansoura University, Mansoura, Egypt; 4 Pediatric Infectious Diseases Unit, Mansoura University Children’s Hospital, Faculty of Medicine, Mansoura University, Mansoura, Egypt

**Keywords:** Acute kidney injury, Multisystem inflammatory disease, MIS-C, COVID-19, Children

## Abstract

**Background:**

Acute kidney injury (AKI) in patients with multisystem inflammatory syndrome (MIS), COVID-19 related infection has been increasingly recognized with a paucity of data on AKI incidence, related mortality, and the requirement of renal replacement therapy in children with MIS (MIS-C).

**Methods:**

This is a retrospective study evaluating the prevalence, severity, management and outcomes of AKI in a cohort of Egyptian children with MIS-children (MIS-C) post-COVID infection. Patients were included if they met the criteria for MIS-C based on CDC guidelines. All patients were evaluated for AKI diagnosis and staging according to the Kidney Disease Improving Global Outcomes (KDIGO) criteria.

**Results:**

Between March 2021 and June 2023, a total of 655 confirmed COVID-19 cases were admitted and then followed up in our hospital, of whom 138 (21%) were diagnosed with MIS-C. Fifty-one patients developed AKI associated with MIS-C post-COVID infection, 42 of whom were included in the analysis. Thirty-one patients had AKI in a formerly healthy kidney, of whom 51% (16 patients) were classified as KDIGO stage 3, 5 patients needed hemodialysis and 13 needed mechanical ventilation. Higher WBCs count, and serum ferritin on admission were associated with more severe AKI (KDIGO stage 3) (*p* = 0.04), while multivariate analysis showed high serum ferritin to be independent predictor of more severe AKI (*p* = 0.02). Two patients (2/31) died during hospital admission, while no residual renal impairment was reported at the time of discharge of patients with previously normal kidney functions.

**Conclusion:**

More than one-third of patients with MIS-C develop AKI. Avoidance of nephrotoxic drugs, early recognition, and prompt management of AKI, including well-timed commencement of dialysis in MIS-C cases, is associated with favorable outcomes.

## Introduction

Since late 2019, a global pandemic known as coronavirus disease-2 (COVID-19), which is caused by severe acute respiratory syndrome coronavirus-2 (SARS-CoV-19), has had a negative impact on people's wellbeing as well as all features of life. The pathophysiology of COVID-19, its effects, and associated disorders have become more obvious over time [1]. Multisystem inflammatory syndrome in children (MIS-C) was initially described in confirmed COVID-19 cases in late April 2020, and by December 2020, the Centers for Disease Control and Prevention (CDC) recorded 1288 cases [2, 3] and defined MIS-C as having persistent fever and inflammatory laboratory markers, as well as signs of severe disease requiring hospitalization and multiorgan involvement (e.g., cardiac, gastrointestinal, renal, hematologic, dermatologic, and neurologic). The affected individual must be less than 21 years old and have had exposure to a confirmed or suspected patient with COVID-19 [4]. The incidence of MIS-C is reported to be nearly 3.16 cases per 10,000 SARS-CoV-2-infected persons [5].

The proportion of pediatric patients with SARS-CoV-2 infection who develop acute kidney injury (AKI) is highly variable between reports [6, 7], with a prevalence in critically ill children approaching 44% [8]. A systematic review lately reported a pooled proportion of patients with MIS-C developing AKI of 20% (11 studies, 4947 patients) [9].

The pathophysiology of renal dysfunction in patients with SARS-CoV-2 infection is multifactorial, including dehydration, poor cardiac output, cytokine storm, direct cytopathic effect of the virus on renal tubular cells and use of nephrotoxic agents [10], whereas in patients presenting with MIS-C, renal hypoperfusion seems to be the major underlying factor for AKI [11, 12].

With insufficient data on AKI incidence, risk factors, effect on mortality, and the required care in children with MIS-C worldwide and the absence of such data from our locality, this retrospective study aimed to evaluate the incidence of AKI, renal pathology, mortality, and the need for kidney replacement therapy (KRT) in MIS-C-related COVID-19 infection for the first time in Egyptian children.

## Materials and methods

A retrospective study of patients with AKI associating MIS-C, related COVID-19 infection admitted to Pediatric Nephrology and Pediatric Intensive Care Units of Mansoura University Children’s Hospital, Mansoura, Egypt. Data from patients admitted and then followed-up between March 2021 and June 2023 were extracted, including 655 cases of COVID infection, of which 51 were diagnosed with AKI associating MIS-C, related COVID-19 infection. All patient data were extracted from the electronic database registry of the hospital. The study protocol was approved by the local Ethics Committee of Mansoura Faculty of Medicine-Institutional Research Board (IRB) (R.23.06.2233). All patients/guardians provided informed consent at the time of hospital admission for possible use of their data in future research.

### Study participants

**Fig. 1:**
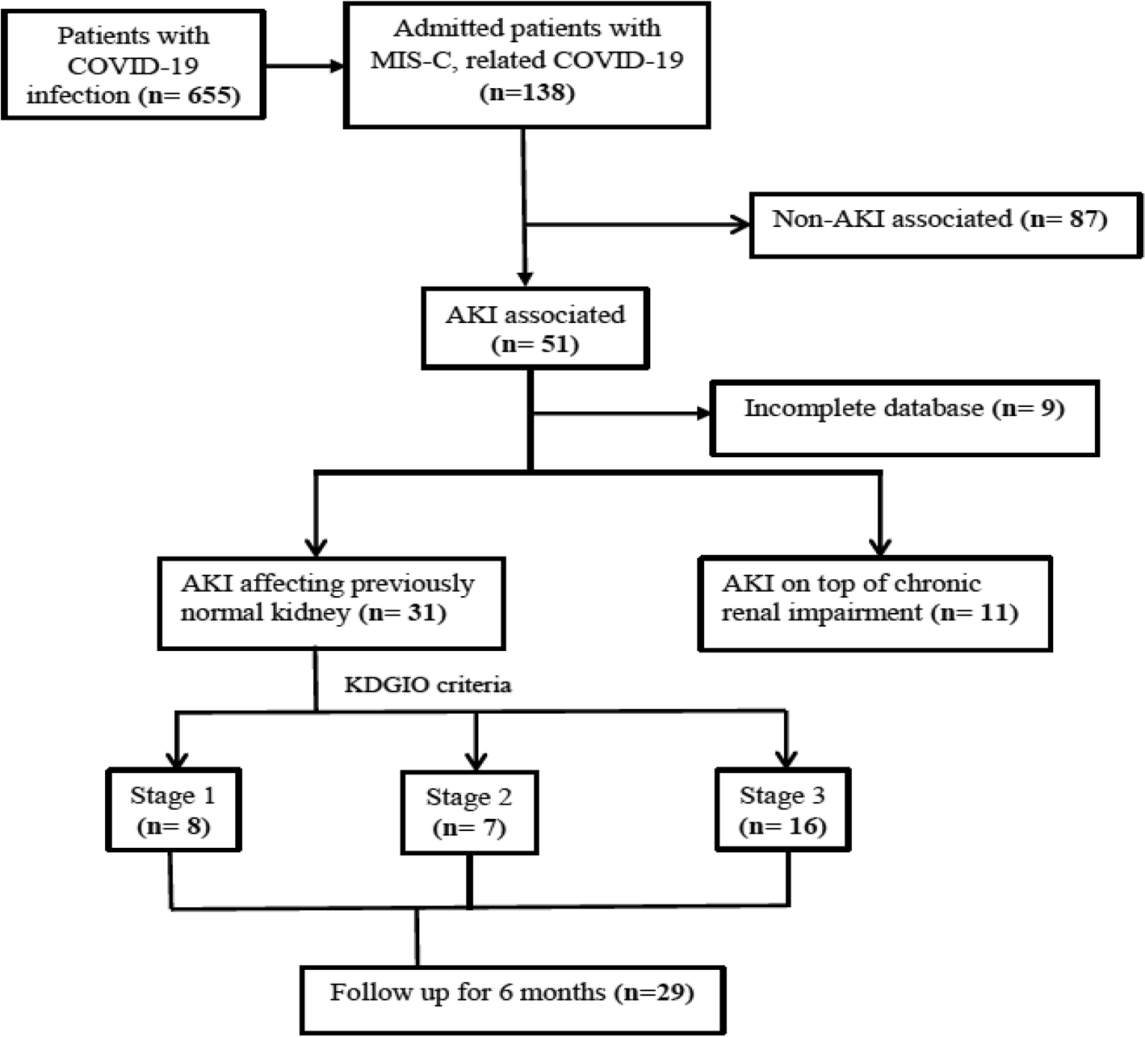
Flow diagram of the study participants. AKI: acute kidney injury, KDIGO: kidney disease improving global outcomes, MIS-C: multisystem inflammatory syndrome in children

Forty-two patients with AKI associating MIS-C, related COVID-19 infection were included in the analysis, 31 with AKI affecting normal kidney and 11 patients had AKI on top of previously diagnosed chronic kidney disease (CKD) (Fig. 1). Out of 138 cases of MIS-C; 47 fulfilled CDC criteria at time of hospital admission while 91 were diagnosed during hospital stay (isolation ward) then were transferred to PICU.

#### Inclusion criteria:

- ◦ Patients with confirmed diagnosis of SARS-CoV-2 by a positive reverse transcriptase-polymerase chain reaction (RT‒PCR) (with or without a positive SARS-CoV-2 antibody test) or positive SARS-CoV-2 serology test only (with negative or unknown RT‒PCR results) were included.
- ◦ The case definition for MIS-C, COVID-19, and related illness was based on the CDC criteria. A confirmed case meets both the clinical and laboratory criteria [13].
- ◦ Definition and staging of AKI (stages 1, 2 or 3) was performed based on the Kidney Disease Improving Global Outcomes (KDIGO)-2012 criteria [14].
- ◦ Patients with AKI developed on top of previously diagnosed CKD were included and analyzed separately.

#### Exclusion criteria:

- ◦ Patients who had a clinical presentation of MIS-C but did not meet the CDC case definition criteria were excluded.

## Methods

### Clinical evaluation

Data extraction included patient demographic features [age, weight, height, body mass index (BMI)] and presenting symptoms. Hospitalization information collected included the duration of hospital stay, level of care needed [pediatric intensive care unit (PICU)/inpatient isolation room], medications received and clinical support needs, including inotropic support, mechanical ventilation, KRT and days to recovery of renal function.

### Biochemical data

Serum creatinine (at admission, peak, and before discharge), complete blood count (CBC), peak values of C-reactive protein (CRP), erythrocyte sedimentation rate (ESR), D-dimer, ferritin, and urinalysis.

### Renal pathology

Percutaneous ultrasound-guided renal biopsy was performed in 3 cases due to persistently elevated serum creatinine despite improvement of general condition, respiratory distress, and inflammatory markers.

### Follow-up

After discharge, all patients were followed in the outpatient clinic for 6 months (after 2 weeks, then monthly). Each follow-up visit included clinical examination, blood pressure monitoring, CBC, urine analysis and serum creatinine assessment.

### Statistical analysis

Data were analyzed with SPSS Statistics (SPSS, IBM Company, Chicago, IL, USA) version 23. The normality of the data was first tested with the Shapiro–Wilk test. Continuous variables were presented as the mean ± SD for parametric data and were compared with Student̕̕ s *t* test or one-way ANOVA test, while for nonparametric data, median (minimum–maximum) was used and Mann‒Whitney or Kruskal‒Wallis tests. Qualitative data are presented as frequencies and percentages. The association between categorical variables was tested using the chi-square test. Kaplan‒Meier curves were plotted for the three stages of AKI to detect the time elapsed until AKI recovery. The* P* value to reject the null hypothesis and consider statistical significance is < 0.05.

## Results

### Participants and descriptive data

Over 24 months, a total of 655 COVID-19 confirmed cases were admitted to our hospital, of which 51 cases developed AKI associating MIS-C post-COVID infection (9 excluded due to incomplete data). The 42 AKI cases included: 11 cases with previously diagnosed CKD (6 cases with unknown primary etiology, 3 cases with steroid-resistant nephrotic syndrome and 2 cases with lupus nephritis) and 31 cases with previously normal kidney functions. For demographic characteristics, patient age, sex and anthropometric measures did not significantly vary between the study groups. Moreover, clinical data, including presenting symptoms, MIS-C associating AKI, vasopressor need and mechanical ventilation use, were not significantly different between the AKI affecting the previously normal kidney group and the CKD group, as summarized in Table 1. Patients with known CKD included 5 patients with CKD stage III, 1 patient with stage IV and 5 patients with stage V. One of those 11 patients died, and 8 continued hemodialysis after discharge.

**Table 1:** Demographic data and clinical features of AKI associated MIS-C, related COVID-19

	**AKI affecting previously normal kidney (*n* = 31)**	**AKI on top of chronic renal impairment (*n* = 11)**	***P*-value**
Age (year) ^a^	7.5 (0.75–17)	9 (5–14)	0.464
Gender ^b^ (Male/female)	19 (61.3%)/12 (38.7%)	6 (54.5%)/ 5 (45.5%)	0.695
Weight (kg) ^a^	23 (7.3–71)	22.5 (13–39)	0.308
Height (cm) ^c^	121.1 ± 31.5	123.5 ± 25.9	0.818
BMI (kg/m^2^) ^c^	18.8 ± 3.8	14.7 ± 1.5	**0.002**
Presenting features ^b^			
Fever	27 (87.1%)	9 (81.8%)	0.667
Dyspnoea	25 (80.6%)	11 (100%)	0.115
GIT symptoms	17 (54.8%)	6 (54.5%)	0.987
Rash	3 (9.7%)	0	0.284
MIS-C associating AKI ^b^			
Cardiac	11 (35.5%)	2 (18.2%)	0.286
Respiratory	25 (80.6%)	11 (100%)	0.115
Gastro-intestinal	9 (29%)	0	**0.044**
Hematologic	23 (74.2%)	7 (63.6%)	0.505
Neurologic	10 (32.3%)	0	**0.031**
Dermatologic	3 (9.7%)	0	0.284
Vasopressors use ^b^	5 (16.1%)	1 (9.1%)	0.567
Oxygen therapy ^b^	15 (48.4%)	9 (81.8%)	0.054
Invasive Mechanical Ventilation b	13 (41.9%)	2 (18.2%)	0.158
Duration of Mechanical Ventilation c	7.3 ± 3.3	8 ± 1.4	0.785
Duration of PICU stay (days)^c^	(*n* = 21). 9.9 $\pm$ 5.2	(*n* = 4). 8.5 $\pm$ 3.1	0.624
Duration of hospital stay (days)^c^	23.6 $\pm$ 12.8	13.5 $\pm$ 9.3	**0.021**

*AKI* Acute kidney injury, *MIS-C* Multi system inflammatory syndrome in children, *PICU* Paediatric intensive care unit, *BMI* Body mass index, *GIT* Gastrointestinal tract. Data presented as median (minimum–maximum) ^a^, number (percent) ^b^ or mean ± SD ^c^ and analysed by Mann–Whitney test ^a^, Chi-Square test ^b^, Student *t*-test ^c^; respectively

### Patients with AKI in previously normal kidney functions

Patients with AKI affecting previously normal kidneys were further categorized according to KDGIO staging into 3 groups. The basic demographic and clinical features of the 3 groups are compared in Table 2. Comorbidities included congenital heart disease (3 cases), B-thalassemia (2 cases), type 1 diabetes (3 cases) and juvenile idiopathic arthritis (1 case).

**Table 2:** Clinical features of AKI patients associated with MIS-C, related COVID

	**AKI stage 1 (*n* = 8)**	**AKI stage 2 (*n* = 7)**	**AKI stage 3 (*n* = 16)**	***P*-value**
Age (year) ^a^	11 (0.75–15)	9.5 (4–17)	7.1 (1–15.3)	0.893
Gender ^b^ Male/Female	4 (50%)/4 (50%)	5 (71.4%)/ 2 (28.6%)	10 (62.5%)/ 6 (37.5%)	0.690
Weight (kg) ^a^	28.5 (7.3–63)	27 (15–57)	23 (9–71)	0.833
Height (cm) ^c^	124.5 ± 38.6	117.7 ± 24.6	120.9 ± 32.1	0.921
BMI (kg/m^2^) ^c^	17.5 ± 4.5	17.8 ± 2.1	19.8 ± 4	0.323
Co-morbid diseases ^b^	1 (12.5%)	1 (14.3%)	7 (43.8%)	0.175
Presenting features ^b^				
Fever	8 (100%)	7 (100%)	12 (75%)	0.116
Dyspnoea	8 (100%)	7 (100%)	10 (62.5%)	**0.031**
GIT symptoms	4 (50%)	3 (42.9%)	10 (62.5%)	0.650
Rash	0	0	3 (18.8%)	0.211
System affection associating AKI ^b^				
Cardiac	4	3	4	0.434
Respiratory	8	7	10	**0.031**
Gastro-intestinal	2	3	4	0.657
Hematologic	6	5	12	0.982
Neurologic	0	3	7	0.077
Dermatologic	0	0	3	0.211
Vasopressors use ^b^	1 (12.5%)	2 (28.6%)	2 (12.5%)	0.596
Oxygen therapy ^b^	5 (62.5%)	5 (71.4%)	5 (31.3%)	0.135
Invasive Mechanical Ventilation ^b^	3 (37.5%)	2 (28.6%)	8 (50%)	0.605
Duration of Mechanical Ventilation(days) ^c^	6.7 ± 4.7	8 ± 1.4	7.4 ± 3.5	0.921

*AKI* Acute kidney injury, *MIS-C* Multi system inflammatory syndrome in children, *PICU* Paediatric intensive care unit, *BMI* Body mass index, *GIT* Gastrointestinal tract. Data expressed as median (minimum–maximum) ^a^, number (percent) ^b^ or mean ± SD ^c^ and analysed by Kruskal–Wallis test ^a^, Chi-Square test ^b^, One-Way ANOVA ^c^; respectively. *AKI* Acute kidney injury, *BMI* Body mass index

**Fig. 2:**
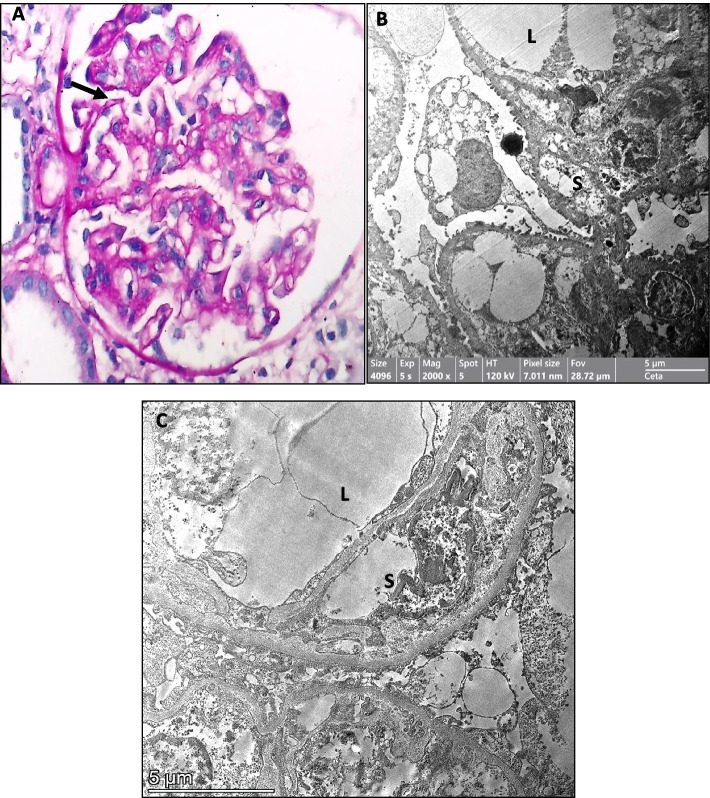
**A** Light microscopy showing a broadened fibrillary mesangium without associated hypercellularity. Some capillaries exhibit a double contoured appearance (→). (PAS stain × 400). **B** and **C**) Electron microscopy showing that the double contoured appearance seen by light microscopy is due to expansion of the subendothelial space by an electron-lucent, fluffy finely granular material with mesangial interposition. (L: capillary lumen, S: subendothelial space divided into subcompartments)

Table 3 demonstrates the laboratory values of stage 1, 2 and 3 AKI patients, CBC on admission and ESR, CRP, ferritin and D. dimer peak values. Admission creatinine levels were significantly higher in stage 3 (*p* = 0.001) than in stages 1 and 2. Higher WBCs count, and serum ferritin on admission were associated with more severe AKI (KDIGO stage 3) (*p* = 0.04), while multivariate analysis showed high serum ferritin to be independent predictor of more severe AKI (*p* = 0.02). Renal biopsy was performed in 3 cases: the first was a concomitantly discovered case of class III lupus nephritis, the second case was a newly discovered CKD stage II, and pathology revealed a chronic stage of diffuse crescentic glomerulonephritis. The third case was a 7-year-old female with AKI in a previously healthy kidney. Figure 2 shows light and electron microscopic findings of the case that revealed the early chronic stage of thrombotic microangiopathy “glomerular type”, focal acute tubular injury and faint IgM deposits.

**Table 3:** Laboratory values of the AKI patients categorized according to KDIGO staging

	**AKI stage 1 (*n* = 8)**	**AKI stage 2 (*n* = 7)**	**AKI stage 3 (*n* = 16)**	***P* value**
WBCs (10^9^/L) ^a^	7.4 (3.7–37.4)	8.1 (7.2–11.5)	20.5 (6.8–51.3)	**0.037**
Neutrophil/Lymphocyte ^a^	5.3 (0.3–13.8)	1.7 (1.2–1.7)	4.9 (0.6–20.2)	0.062
Haemoglobin (g/dl) ^b^	8.9 ± 1.6	9.4 ± 0.5	8.5 ± 3.2	0.759
Platelets (10^9^/L) ^b^	122 ± 87	393 ± 129	241 ± 124	**0.001**
C-reactive protein (mg/L) ^a^	9 (6–24)	48 (6–96)	36 (6–96)	**0.041**
ESR (mm/hour) ^a^	20 (10–40)	15 (8–82)	67 (28–95)	**0.005**
Serum Ferritin (ng/mL) ^a^	309 (77–703)	315 (19–721)	712 (196–1899)	**0.04**
D-Dimer (mg/L) ^a^	3.9 (5–8.5)	2.4 (2–3.6)	1.1 (3–8)	0.302
Admission serum creatinine (mg/dl) ^b^	1.1 ± 0.4	1.6 ± 0.9	5.8 ± 3.2	**0.001**
Peak serum creatinine (mg/dl) ^b^	1.8 ± 0.1	2.4 ± 0.2	8.4 ± 3.9	**0.001**
Urine examination ^c^				
RBCs (≥ 5/HPF)	8 (100%)	7 (100%)	11 (68.8%)	0.061
Protein (≥ 1 +)	4 (50%)	4 (57.1)	11 (68.8%)	0.652

*AKI* acute kidney injury, *KDIGO* kidney diseases improving global outcomes.Data expressed as median (minimum–maximum) ^a^, mean ± SD ^b^ or number (percent) ^c^ and analysed by Kruskal–Wallis test ^a^, One-Way ANOVA ^b^, Chi-Square test ^c^; respectively. *AKI* Acute kidney injury, *EBCs* White blood cells, *RBCs* Red blood cells, *HPF* High power field, *ESR* Erythrocyte sedimentation rate

**Fig. 3:**
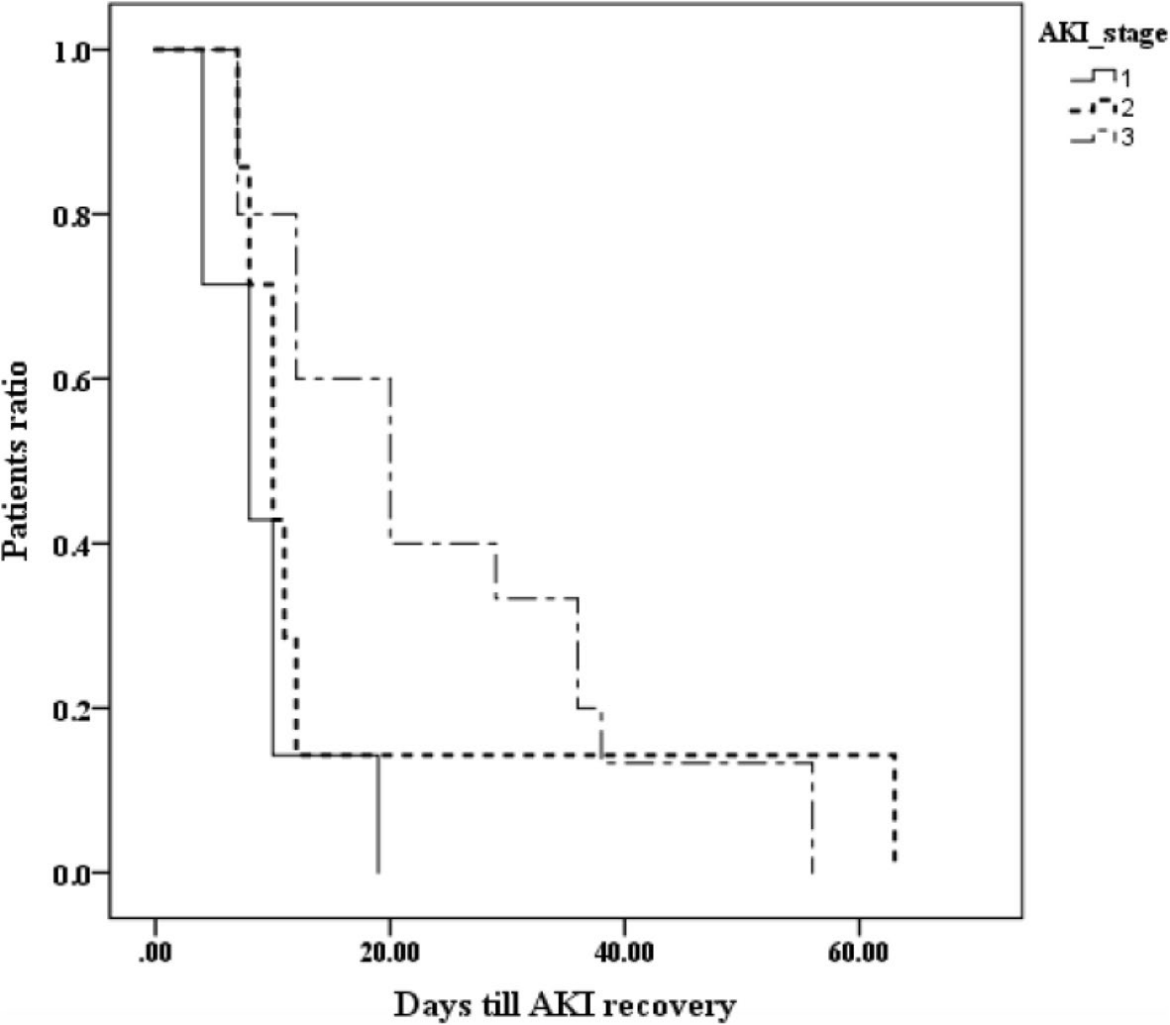
Kaplan‒Meier curve for time to AKI recovery as categorized by KDIGO staging

All patients received intravenous immunoglobulin (IVIG) at a dose of 2 g/kg, which was repeated after 48 h if fever persisted, cardiac affection, organ dysfunction deteriorated, or inflammatory markers worsened otherwise corticosteroids was considered. Only 2 patients received favipiravir (due to limited availability and oral route which was not appropriate for several cases with GIT involvement). Hemodialysis was initiated in 5 cases (due to volume overload, refractory metabolic acidosis or uremia), and the number of dialysis sessions ranged between 5–12 sessions. In Table 4, a significantly higher duration of hospital admission was observed in Stage II (29.3 ± 15.3 days; *p* = 0.018), with the longest duration of PICU stay also reported in stage 3 (20 days), but with no statistically significant difference in PICU stay reported between AKI stages. Days elapsed for resolution of AKI were significantly higher in stage 3 [20 (7–56) when compared with stage 1 [8 (4–19) and stage 2 [10 (7–63); (*p* = 0.034) (Fig. 3).

**Table 4:** Outcome parameters of the AKI patients categorized according to KDIGO staging

	**AKI stage 1 (*n* = 8)**	**AKI stage 2 (*n* = 7)**	**AKI stage 3 (*n* = 16)**	***p*-value**
Length of hospital stay (days) ^a^	13.1 + 5.6	29.3 + 15.3	26.3 + 11.7	0.018
Length of PICU stay (days) ^c^	(*n* = 4) 6(2–18)	(*n* = 7) 10(5–15)	(*n* = 10) 9.5(2–20)	0.505
Haemodialysis ^b^	0	0	5	0.061
Days for resolution of AKI ^c^	(*n* = 7) 8 (4–19)	(*n* = 7) 10 (7–63)	(*n* = 15) 20 (7–56)	0.034
Death ^b^	1	0	1	0.616

*AKI* acute kidney injury, *KDIGO* kidney diseases improving global outcomes, *PICU* pediatric intensive care unit. Data expressed as mean ± SD ^a^, number (percent) ^b^ or median (minimum–maximum) ^c^ and analysed by One-Way ANOVA ^a^, Chi-Square test ^b^, Kruskal–Wallis test ^c^; respectively

Two patients deceased during hospital admission. The first was diagnosed with dilated cardiomyopathy (familial) and died with extensive pneumonia, while the second patient had ARDS, pancytopenia, autoimmune encephalitis, acquired secondary bacterial infection and septic shock.

## Discussion

At the beginning of the pandemic, children were considered to be at lower risk of COVID-19, with a lower incidence of infection and milder clinical course [15]**.** Later, small cohort studies and case reports highlighted kidney involvement in children with COVID-19 infection [16, 17]**.** There have also been few reports supporting a high risk of AKI occurrence in MIS-C, related COVID-19 infection [9] however, there has been no consensus regarding the accurate incidence, outcomes, and related mortality among this population. Therefore, our study was executed to address the prevalence of AKI in children with post-COVID MIS-C and to assess severity and outcome in different stages.

The reported incidence of AKI in children with COVID-19 infection is highly variable between studies. In the current study, 51 patients were diagnosed with AKI, which represented 37% of patients with MIS-C-related COVID-19 and 7.8% of total COVID-19 patients admitted to our hospital during the study period. Grewal et al., reported AKI in 24% of total COVID cases, with a differential incidence of 54% among MIS-C patients and only 9% in those with acute SARS-CoV-2 infection without MIS-C [12]**,** and in a recent systematic review, AKI was reported in 20% of MIS-C patients [9]**.** This variability could be related to variances in AKI definitions applied, rates of hospitalization, concomitant comorbid factors, and incidence of MIS-C cases. A systematic review reported that the incidence of AKI was also higher in studies that used the KDIGO definition of AKI (24%) than in studies that used alternative definitions of AKI (18%), and there was a slightly higher proportion of children developing AKI in single-center studies (23%) than in multicenter studies (20%) [9]**.** The incidence of AKI in pediatric patients is variable, ranging from 5–37% in the PICU with variable requirements for KRT; therefore, patients with MIS-C have a comparable risk of AKI when compared to patients in the PICU due to other causes [18, 19].

The mechanism of AKI in SARS-CoV-2 patients is multifactorial, including dehydration, poor cardiac output, cytokine storm and microvascular thrombosis [20] as well as the direct cytopathic effect of the virus on renal tubular cells due to the presence of angiotensin-converting enzyme 2 receptors, which serve as an entrance door for SARS-CoV-2, and the use of nephrotoxic drugs. However, the mechanism implicated in AKI development in MIS-C patients is chiefly due to renal hypoperfusion because those patients had longer durations of ICU stay, were more susceptible to hypoxemia, particularly if they developed ARDS [21], or needed more inotropic support and had higher markers of cardiac dysfunction compared to acute SARS-CoV-2-AKI patients [22, 23].

Fever and dyspnea were the most common presenting features in our patients (36/42, 85.7%, each). In addition, more than 30% of patients with stage 3 AKI needed oxygen therapy, and 50% needed invasive mechanical ventilation, which may support the role of hypoxemia in AKI pathogenesis. This is consistent with the findings of Sabaghian and colleagues, who reported a statistically significant increase in the number of cases with respiratory system involvement in adults with stage 3 AKI [24].

Different comorbidities have been reported to be associated with the development and worsening of AKI in MIS and COVID-19 infection in both adults and children. Grewal et al. reported that nearly 60% of children in the AKI group had underlying comorbidities: pulmonary (18%), neurological (8%), malignancies (7%), sickle cell disease (5%), hypertension (3%), immunosuppression (2%), type 1 (2%), type 2 diabetes mellitus (2%), and cardiac conditions (1%) [12]*.* Adult studies reported that the most common comorbidities were diabetes, hypertension and hyperlipidemia that were documented in 40%, 61.4% and 57.1% of patients with COVID-19 and AKI, respectively [25], while a history of previously diagnosed CKD was reported in 22.2% of patients [23].

Kidney biopsy was performed in 3 cases in our cohort. Recognizing and properly diagnosing renal involvement in patients with COVID-19 could be challenging (due to the lack of direct proof of viral infection, e.g., viral particles) and requires a proper integration of clinical and pathological data [26]. A wide range of renal pathological findings have been described to associate COVID-19 infection in adults both in native and transplanted kidneys, such as collapsing glomerulopathy (CG) and other forms of focal segmental glomerulosclerosis, acute tubular necrosis (ATN), IgA nephropathy, thrombotic microangiopathy, crescentic glomerulonephritis, minimal change disease, membranous nephropathy, and anti-glomerular basement membrane disease [27–33]. SARS-CoV-2 antigens have been detected by immunohistochemistry in kidney tubules, and virus particles were detected in the renal tubular epithelium and podocytes by transmission electronic microscopy [34]. COVID-19-related immune thrombosis is due to macrophage activation and cytokine storms leading to elevated CRP, ferritin and D-dimer levels, which are correlated with worse outcomes [35]. Scarce renal pathological reports in children with COVID-19 infections are available including minimal change disease, C3 glomerulopathy [26], acute necrotizing glomerulonephritis [36], diffuse and segmental mesangial-proliferative glomerulonephritis and acute tubule-interstitial nephritis [37].

Stage 3 AKI patients reported significantly higher WBCs count, ESR, serum ferritin and creatinine on admission, while no significant difference was detected between AKI stages regarding age, associated comorbid diseases, need for inotropes and mechanical ventilation. Tastemel Ozturk et al. reported a significant relationship between older age and AKI in MIS-C patients in univariate analysis, which was lost in multivariate analysis [38]. The median age of patients with AKI was more likely to be younger than that of patients without AKI (9 years vs. 10.5 years; *p* = 0.08) [39]. Higher values of inflammatory biomarkers, such as WBCs, CRP, procalcitonin, D-dimer, ferritin and IL-6, were observed in MIS-C patients presenting with AKI. In the same studies associated with systolic dysfunction, the need for inotropes and lower levels of albumin and bicarbonates were monitored. Therefore, these data suggest a double component in the pathogenesis of AKI in MIS-C due to both an inflammatory pathway and prerenal injury [40–42].

Hemodialysis (conventional and hemodiafiltration) was initiated in 5 patients (AKI stage 3), and 5–12 sessions were performed per patient. Unfortunately, continuous renal replacement therapy (CRRT) was not available at our institution at that time, so intermittent dialysis was used. KRT in the setting of multiorgan disease syndrome should be initiated with expertise. KRT can be utilized non-selectively to clear inflammatory mediators via convection, adsorption and diffusion. CRRT corrects fluid overload and manages solute levels to provide hemodynamic stability in catabolic pediatric patients [43]. Immediate initiation of preemptive KRT in cases with progressive symptomatic respiratory insufficiency improves overall outcomes. The pediatric CRRT group advises the early initiation of KRT in critically ill COVID-19-related immune dysregulation syndrome patients as it has been shown to mitigate fluid overload, enhance cytokine clearance, improve oxygenation and create hemodynamic stability earlier, leading to better overall outcomes, but if CRRT is not available, intermittent hemodialysis is satisfactory [44].

The mean duration of PICU stay for patients with AKI with previously normal kidney function was 9.9 days, with the longest duration reported in AKI stage 3 (20 days). Grewal et al. reported that all patients in the MISC-AKI group needed admission to the PICU with a median duration of PICU stay in patients with AKI of 7.5 days [12], while Özen et al. (2023) reported a length of stay in the PICU of 8 days [45]. These differences may be related to differences in the severity of cases and standards for PICU admission and discharge.

Only 2 mortalities were reported in our cohort, representing 4.8% of 42 cases with AKI, 1.4% of 138 MISC-related COVID-19 cases and 0.3% of total COVID-19 cases admitted over 2 years. A report from North America reported a 7.7% mortality rate among 274 pediatric COVID patients with AKI [46], while a report from Saudi Arabia stated that AKI was significantly associated with mortality (42% vs. 0%), compared to patients with normal kidney function, and after adjustment for age, sex, and the presence of comorbidities, AKI was still drastically associated with mortality [7]. Bjornstad et al., a multicenter study throughout the United States, Eastern Europe, and Russia, reported a mortality rate of 6% among AKI COVID-19 pediatric patients and 5% among pediatric COVID-19 patients without AKI [16].

AKI is considered an independent risk factor for increased mortality in critically ill patients with any disease [47]. Kidney involvement has also been reported as an indicator of poor prognosis irrespective of initial COVID-19 severity [48]*,* yielding avoidance of nephrotoxic drugs, early detection and prompt management of renal function abnormalities improve the prognosis, which can explain the low mortality rates in our cohort. Additionally, no residual renal impairment was reported in 29 children (with previously normal kidney function) at the time of discharge, and all had normal urine analysis and serum creatinine during the 6-month follow-up period. This is inconsistent with the findings of Kari et al., who reported that 9% of their study population had residual renal impairment at the time of discharge and that factors associated with residual renal impairment were decreased tissue perfusion, sepsis, worsening clinical condition, or comorbidities [7]. Tastemel Ozturk et al. reported AKI recovery at discharge to be 63.6% in COVID-19 survivors and 100% in MIS-C patients [38].

### Strengths and limitations

This is the first study to present the epidemiological features and outcomes of AKI in Egyptian children with MIS-C-related COVID-19 infection***.*** The limitations of the study include the limited number of subjects (owing to the percentage of MISC among COVID children), being a single center study, the retrospective nature of the study, the changes in virus virulence and emergence of new variants in this process due to the long period in which the patients were included, and the changes in the treatments applied and not including a non-AKI group for comparison.

## Conclusion

AKI can occur in more than one-third of MIS-C cases admitted to the PICU, with higher WBC count, ESR, and serum ferritin on admission associated with more severe AKI. Avoidance of nephrotoxic drugs, early recognition, and prompt management of renal impairment in MIS-C cases, including well-timed initiation of dialysis, are associated with favorable prognosis.

## Data Availability

The datasets used and/or analysed during the current study are available from the corresponding author on reasonable request.

## Abbreviations

AKI: Acute kidney injury
ARDS: Acute respiratory distress syndrome
CDC: Centers for Disease Control and Prevention
CKD: Chronic kidney disease.
COVID-19: Coronavirus Disease-2
CRRT: Continuous renal replacement therapy
HD: Hemodialysis
KDIGO: Kidney Disease Improving Global Outcomes
MIS-C: Multisystem inflammatory syndrome in children
PCR: Polymerase chain reaction.
PICU: Pediatric intensive care unit
KRT: Kidney replacement therapy
SARS-CoV-19: Severe acute respiratory syndrome coronavirus-2
